# An Innovative Vortex-Assisted Liquid-Liquid Microextraction Approach Using Deep Eutectic Solvent: Application for the Spectrofluorometric Determination of Rhodamine B in Water, Food and Cosmetic Samples

**DOI:** 10.3390/molecules29143397

**Published:** 2024-07-19

**Authors:** Sofia Kakalejčíková, Yaroslav Bazeľ, Van Anh Le Thi, Maksym Fizer

**Affiliations:** 1 Department of Analytical Chemistry, Institute of Chemistry, Pavol Jozef Šafárik University in Košice, 040 01 Košice, Slovakia; sofia.kakalejcikova@student.upjs.sk (S.K.); van.anh.le.thi@student.upjs.sk (V.A.L.T.); 2 Department of Chemistry, University of Nevada, Reno, 1664 N. Virginia Street, Reno, NV 89557-0216, USA; mmfizer@gmail.com

**Keywords:** deep eutectic solvents, electrostatic potential, fluorescence detection, Hansen solubility parameters, non-covalent interactions, rhodamine B, vortex-assisted liquid–liquid microextraction

## Abstract

A new green and highly sensitive method for the determination of rhodamine B (RhB) by deep eutectic solvent-based vortex-assisted liquid–liquid microextraction with fluorescence detection (DES-VALLME-FLD) was developed. The extraction efficiency of conventional solvents and different deep eutectic solvent (DES) systems composed of tetrabutylammonium bromide (TBAB) and an alcohol (hexanol, octanol, or decanol) in different ratios were compared. DFT calculations of intermolecular electrostatic and non-covalent interactions of the most stable RhB forms with DES and water explain the experimental DESs’ extraction efficiency. Semiempirical PM7 computations were used to obtain Hansen solubility parameters, which supported the good solubility of the monocationic RhB form in selected DESs. The dependence of the linear calibration of microextraction into 100 µL DES was observed in the RhB calibration range from 0.2 to 10.0 µg L^−1^ with a correlation coefficient of R^2^ = 0.9991. The LOD value was calculated to be 0.023 µg L^−1^. The accuracy and precision of the proposed method were verified over two days with RSD values of 2.9 to 4.1% and recovery of 94.6 to 103.7%. The developed method was applied to the determination of RhB in real samples (tap water, energy drink, and lipstick).

## 1. Introduction

Rhodamine B (RhB) is a water-soluble and inexpensive basic red dye belonging to the class of xanthenes. It is widely used as a dye in textiles and food and is also known as a fluorescent water indicator [1]. Due to its high stability, excellent tinting abilities, and cheap cost, it is preferred over natural dyes [2]. However, irritant, carcinogenic, chronic, genotoxic, neurotoxic, and toxic effects of RhB dye have been observed in humans and animals. Due to these serious adverse effects of RhB on humans and animals, its use in cosmetic and food products has been banned in many countries [3,4,5,6,7,8]. Nevertheless, RhB dye is illegally used because of its low cost, stability, chemical and light resistance, and high efficiency. Therefore, accurate, sensitive, and reliable RhB determination is very important in ensuring safe water and food supplies and protecting human health [9].

Analytical methods commonly used for the determination of RhB are UV–Vis spectrophotometry [10,11,12], high-performance liquid chromatography [13], colorimetry [14], voltammetry [15], etc. Spectrofluorimetry is a fast, sensitive, and relatively simple analytical method, but only a few fluorescent methods for determining RhB can be found in the literature, primarily based on the use of micelles [16,17], magnetic nanoparticles [18], and solid-phase extraction [19,20].

Different methods are used for separation and preconcentration, such as microextraction, which is often combined with analytical detection techniques nowadays. These resulting combinations are ecological since they reduce or eliminate the use of hazardous organic solvents. The sample volume and the amount of organic solvent can be decreased using liquid-phase microextraction (LPME) [21]. LPME procedures are compact, easy to use, and inexpensive due to their ability to transfer analytes from an aqueous sample using microliter quantities of organic solvents [22]. The choice of extraction solvent significantly affects the selectivity and efficiency of the method [23]. However, the use of organic solvents does not follow the principles of green analytical chemistry [24] and green sample preparation [25], as they are hazardous to the environment and to the analyst. In recent years, the main development trend of liquid-phase microextraction is the introduction of new green solvents as extraction agents to eliminate traditional organic solvents, reduce environmental pressure, and improve the green stage of pretreatment [26]. In this way, several alternative solvents, such as ionic liquids [23], supramolecular solvents [27], switchable hydrophilicity solvents [28], and deep eutectic solvents (DES) [29,30] are increasingly used in LPME techniques.

DESs are among the most promising environmentally friendly solvents obtainable today for the extraction and preconcentration of analytes from various matrices. These solvents minimize the hazards, expense, and negative impacts and risks on the environment associated with traditional solvents. Moreover, they prevent the components from degrading, which would otherwise occur at the high temperatures required to extract toxic organic solvents [31,32,33]. The primary advantages of using DESs include their straightforward preparation and broad versatility, stemming from a diverse array of components and their potential combinations. Furthermore, DESs demonstrate exceptional chemical and thermal stability, varying viscosity, and minimal volatility at room temperature. Their remarkable solvent properties enable the effective dissolution of diverse analytes, from small molecules to macromolecules, facilitated by their proton-donating or -accepting capabilities and ability to form hydrogen bonds with various analyte types [34]. Overall, DESs have exhibited significant potential in extracting a wide range of analytes across numerous fields, including the analysis of food and environmental samples [33,34,35]. The liquid-phase microextraction method using a deep eutectic solvent combined with fluorescence detection for the RhB determination has not yet been described in the literature.

In this study, a new green and highly sensitive method for the determination of RhB based on deep eutectic solvent-based vortex-assisted liquid–liquid microextraction with fluorescence detection (DES-VALLME-FLD) was proposed. The method was successfully applied to real samples such as tap water, lipstick, and an energy drink.

## 2. Results and Discussion

### 2.1. Reaction Chemistry and Effect of pH

RhB has the structure of an elongated carbon chain on both sides of the benzene ring, with nitrogen in the middle, together with a carboxyl group [36,37]. RhB is thought to exist in solutions in three forms: the cation (R^+^), the zwitterion (R^+−^), and the colorless lactone (R^0^) [38]. The dissociation constant (pK) of the dye is 3.20, which means that in solutions with pH > 3.0–4.0, the dissociated form corresponding to the zwitterion should be dominant. An important feature of the RhB dye is that the absorption and fluorescence spectra of the R^+^ and R^+−^ forms are similar in the aqueous environment.

**Figure 1:**
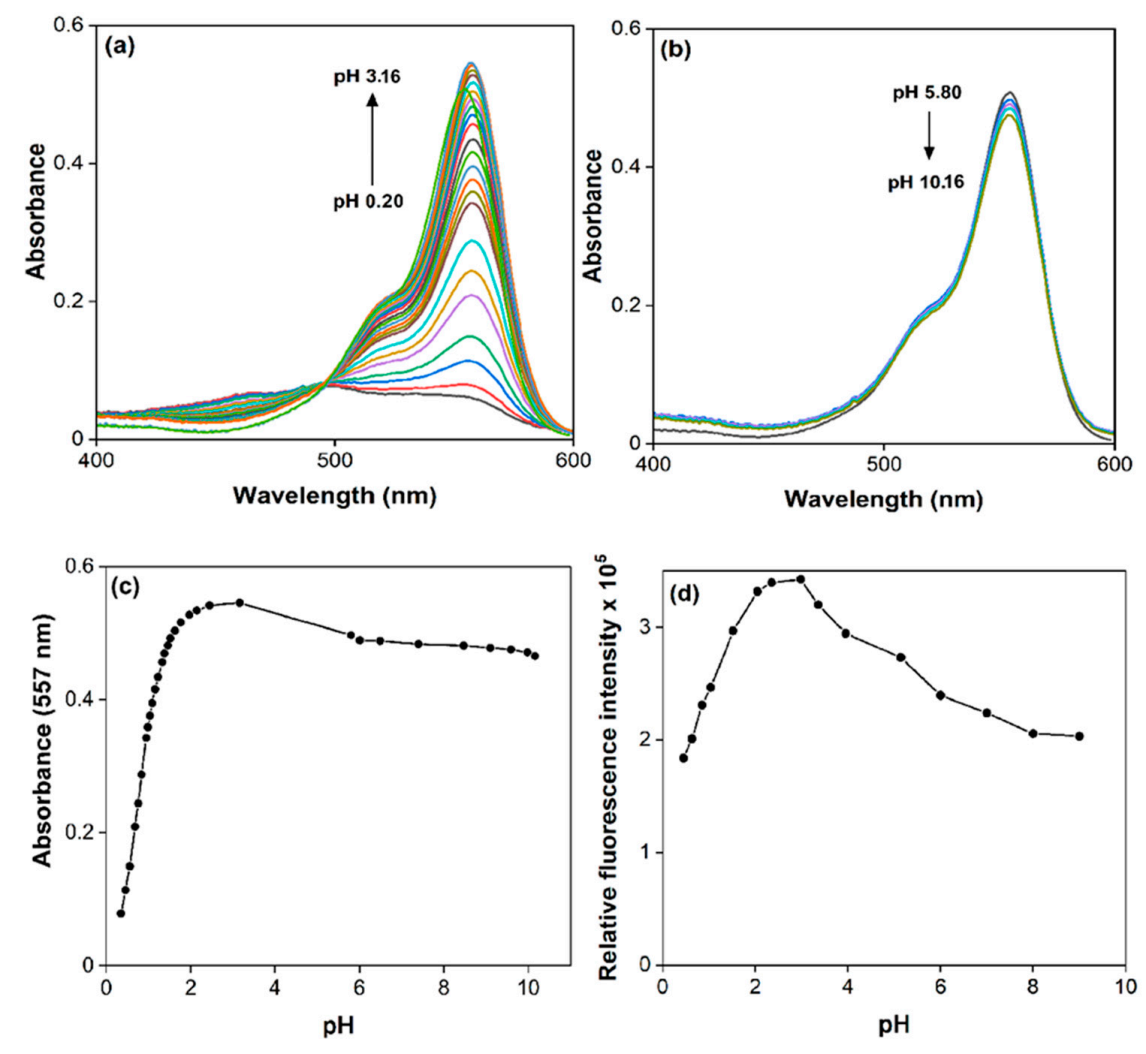
Influence of pH on the absorbance (**a**–**c**) and extraction (**d**) of RhB. c (RhB) = 2.5 × 10^−5^ M (**a**–**c**) and 1 × 10^−8^ M (**d**), TBAB:hexanol (1:3) = 100 µL.

The maximum absorbance of aqueous dye solutions (pH ≥ 5.80) is 553 nm; slight acidification of the aqueous phase with HCl solution shifts the maximum absorbance to 557 nm (Figure 1a). An increase in the pH value and the transition to an alkaline environment barely affects the spectral properties of the agent, since the maximum absorbance remains unchanged at 553 nm in the pH range 5.80–10.16 (Figure 1b). This can be explained by the similarity of the chromophore system R^+^ and R^+−^ forms. The effect of the charge of the 2′-substituent from the dissociation of the carboxylate group is negligible and causes only a small bathochromic shift. The tautomeric equilibrium between the zwitterion and the lactone strongly depends on the solvent, primarily on its ability to form hydrogen bonds with the dye [39,40,41,42]. Therefore, in polar solvents (including water), dyes are found in the colored zwitterion form, whereas, in aprotic solvents, the colorless lactone usually dominates. The proportion of the colorless lactone form of dye R^0^ in aqueous solutions is negligibly small. However, RhB dye can also be in another form—protonated (HR^2+^). The process of protonation of the dye was minimally studied, and the authors could not find a reliable value of the protonation constant in the literature. Therefore, the authors examined the state of the dye in acidic solutions in more detail. A suitable tool for this was the procedure previously reported by our team [43,44], which enables simultaneous in situ control of the pH of the solution and the spectral properties of the RhB solution using a standard optical probe, with the necessary accessories for connection to a spectrophotometer using a submerged glass pH electrode. Before the experiment itself, the linearity interval of the calibration dependence of the absorbance (at λ_max_ = 553 nm) on the dye concentration was investigated. The linearity of the calibration dependence in the RhB concentration range 0–2 × 10^−5^ M is described by the equation y = 0.0438x − 0.0002 (y represents absorbance, x represents concentration of RhB in mg L^−1^), with a correlation coefficient of 0.9978.

The protolytic properties of the dye were studied by the spectrophotometric titration procedure. After each addition of hydrochloric acid, the pH value and the UV–Vis spectrum were measured simultaneously in the range of 400–600 nm, while the absorbance at λ_max_ was adjusted by the volume dilution factor. As can be seen in Figure 1a, the absorption spectra at different pH values in the pH interval 0.20–5.80 intersect at the isosbestic point of 496 nm. This may indicate an equilibrium between the two cationic forms of the dye. The cationic form R^+^ dominates in a solution with pH ≥ 2.0 and is characterized by an intense absorption maximum at 557 nm. The protonated form of the dye HR^2+^ dominates in strongly acidic solutions with pH < 1.0 and is characterized by a low absorption intensity at λ_max_ of 460 nm. The calculated value of the protolytic constant, according to the data shown in Figure 1c, is a pKp of 0.76. Probably, in strongly acidic solutions, protonation of the dye occurs on the N atom of the amino group, and this significantly disrupts the chromophoric system of the dye, which is manifested by a decrease in its coloring intensity.

The aforementioned equilibrium processes also have an impact on the extraction of RhB. As can be seen from Figure 1d, the maximum extraction of RhB, using a DES composed of tetrabutylammonium bromide (TBAB):hexanol in a molar ratio of 1:3, is observed in an acidic environment, in the range of pH 2.05–3.20. As the acidity decreases, the dye extraction decreases, and in the pH range of 8.06–9.20, the fluorescence intensity reaches less than 60% of the maximum intensity. The decrease in extraction in strongly acidic solutions is even more significant; in the pH range of 2.05–0.45, the decrease in extraction takes place almost linearly up to 50% of the maximum intensity. Comparisons of the effect of pH on the state of the dye in aqueous solutions and its extraction are generally in agreement. It is assumed that a DES best extracts the cationic, undissociated form of RhB. The protonated form of the HR^2+^ dye, which dominates in strongly acidic solutions with pH < 1.0, and the zwitterion, which dominates in aqueous solutions with pH > 3.0, are not extracted by the DES. In other studies, pH 3.0 was used as the optimal value.

### 2.2. Investigation of the Experimental Conditions

Various factors influencing the relative fluorescence intensity were studied. The optimization was mainly focused on investigating the influence of chemical characteristics, which are described in more detail in the following subsections. Other factors examined included mixing and vortexing time, as well as speed and effects of centrifugation.

#### 2.2.1. Effect of DES

One of the most commonly used DESs for analytical applications is TBAB-based DESs, which act as hydrogen bond acceptors and are most commonly combined with various hydrogen bond donors, such as long-chain alcohols or carboxylic acids. The authors of [45,46] found that DESs synthesized from TBAB and long-chain alcohols (from amyl alcohol to dodecanol) decompose in the aqueous phase so that the alcohol can act as an extraction solvent and TBAB as a dispersing agent, which facilitates mass transfer between the aqueous and organic phases. This provides a good understanding of why such DESs have proven to be effective for the preconcentration and separation of many analytes, including rhodamine dyes [11,12,47]. Therefore, we chose similar extraction systems for our study.

A suitable extraction solvent should primarily be slightly soluble in water, have a high extraction efficiency for the target analyte, and, at the same time, meet the requirements of green analytical chemistry. DESs, hexanol, decanol, n-amyl acetate, octanol, toluene, chloroform, and tetrachloromethane were tested as extraction agents.

**Figure 2:**
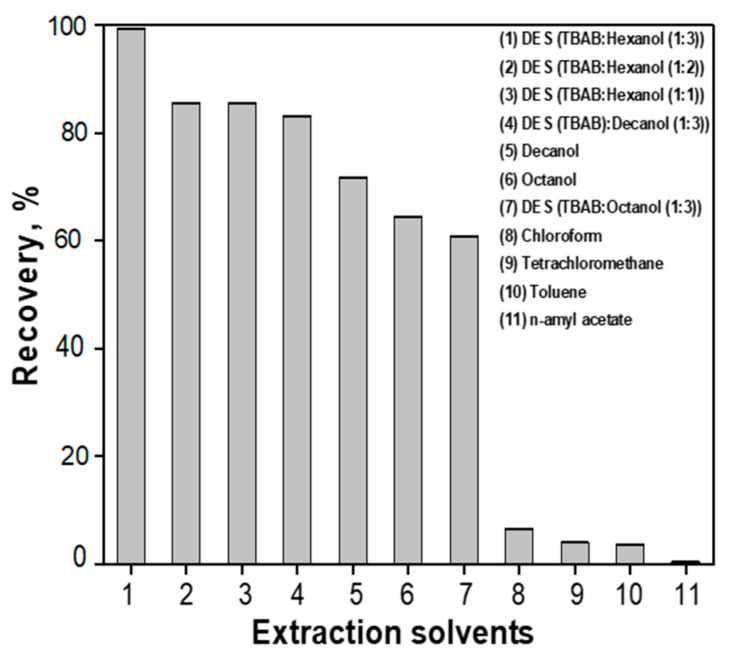
Effect of extraction solvents on the relative fluorescence intensity. c (RhB) = 2 × 10^−7^ M, pH = 3.0.

From the results shown in Figure 2, it can be seen that the DES composed of TBAB:hexanol in a molar ratio of 1:3 achieved the highest yield value. Other DESs showed lower extraction efficiency, while tetrachloromethane, toluene, and n-amyl acetate showed low extraction efficiency. From the obtained results, the DES composed of TBAB:hexanol in a molar ratio of 1:3 was chosen as the most suitable extraction agent to meet the requirements of green analytical chemistry due to its low toxicity.

The observed highest extraction efficiency of the DES composed of TBAB:hexanol in a molar ratio of 1:3 can be explained by estimating its extraction/solvation ability, which, in turn, can be approximately estimated by considering the extraction/solvation ability of its components. Adding TBAB to hexanol increases its polarity compared to the free alcohol molecule—this will be shown in the next section by analyzing electrostatic potential (see Figure 5). On the other hand, the increase of the number of hexanol molecules from 1 to 3 (changing the TBAB:hexanol ratios from 1:1 to 1:2 and then to 1:3) leads to a significant reduction in the electron donating site of the DES from −121 kcal/mol, to −115 kcal/mol and then to −107 kcal/mol (see Figure 5). Considering the known principle “like dissolves like”, we assume that the addition of one part of TBAB to three parts of 1-hexanol forms a mixture whose polarity is very similar to that of RhB. Here, we should mention that a good polarity criterion is logPo/w [48], which, in the case of RhB, is estimated to be about 1.34–1.78 [49,50]. In the case of alcohols, logP equals 2.03 (hexanol), 3.00 (octanol), and 4.57 (decanol) [51]. Among the considered alcohols, hexanol’s logP is the closest to that of RhB. Adding one part of TBAB to three parts of hexanol will increase its polarity (virtually, this would decrease logP) to an optimal value.

#### 2.2.2. Effect of Vortex Mixing and Its Optimization

The effect of vortexing time was optimized in the range from 0 to 60 s at a speed of 2000 rpm. The maximum fluorescence intensity occurred after 15 s of vortexing, with the signal remaining constant between 15 to 30 s, followed by a gradual decrease in relative fluorescence intensity after 30 s of vortexing.

The speed of vortexing was optimized in the interval from 1200 to 3000 rpm. The maximum relative fluorescence intensity was reached at 2000 rpm. After reaching the maximum relative fluorescence intensity, a gradual decrease in signal intensity influenced by an increase in the swirling speed was noticed. Therefore, a vortexing time of 15 s and a vortexing speed of 2000 rpm were chosen for the following experiments.

#### 2.2.3. Effect of Centrifugation and Its Optimization

The time and speed of centrifugation are important and necessary parameters in the optimization of extraction conditions. The centrifugation process was investigated simultaneously using centrifugation speed and time between 1000 to 4000 rpm and for 1 to 5 min. Based on the results, it was found that the centrifugation process must be applied for at least 3 min at 1500 rpm. These centrifugation parameters were adopted as optimal conditions, and subsequent microextractions were performed using these optimal conditions.

### 2.3. Interference Study

The effect of interfering substances was studied under optimal conditions with a constant concentration of RhB and different concentrations of interfering ions. Substances that caused an error of less than ±5% in the measurement of analytical signals did not affect the determination of RhB.

Most of the investigated ions, typically present in tap water, do not seriously interfere with the determination of RhB. Experimental results showed that Br^−^, Cl^−^, F^−^, I^−^, NO_3_^−^, SCN^−^, CO_3_^2−^, H_2_PO_4_^−^, Zn^2+^, NH_4_^+^, K^+^, Al^3+^, Mg^2+^, Na^+^, Mn^2+^, Ca^2+^, Fe^3+^, and Ni^2+^ did not interfere in RhB measurements up to concentrations of 0.01 mol L^−1^, while Cr^6+^ and SO_3_^2−^ did not impact measurements up to 5.0 mmol L^−1^, and NO_2_^−^, Cu^2+^, Co^2+^, and Ba^2+^ were tolerated up to 1.0 mmol L^−1^. Sodium dodecyl sulfate and Triton X-114 are examples of anionic and nonionic surfactants that do not interfere at concentrations of 0.1 mmol L^−1^ and 0.01 mol L^−1^. Cetylpyridinium chloride is a cationic surfactant that can start interfering with RhB at concentrations higher than 1.0 μmol L^−1^. A further source of interference is the extraction of different dyes simultaneously. Fluorescein is tolerated up to 1.0 μmol L^−1^, Eriochrome Black T and erythrosine up to 0.5 μmol L^−1^, and rhodamine 6G at 0.1 μmol L^−1^.

### 2.4. Analytical Characteristics of the DES-VALLME-FLD Method

**Figure 3:**
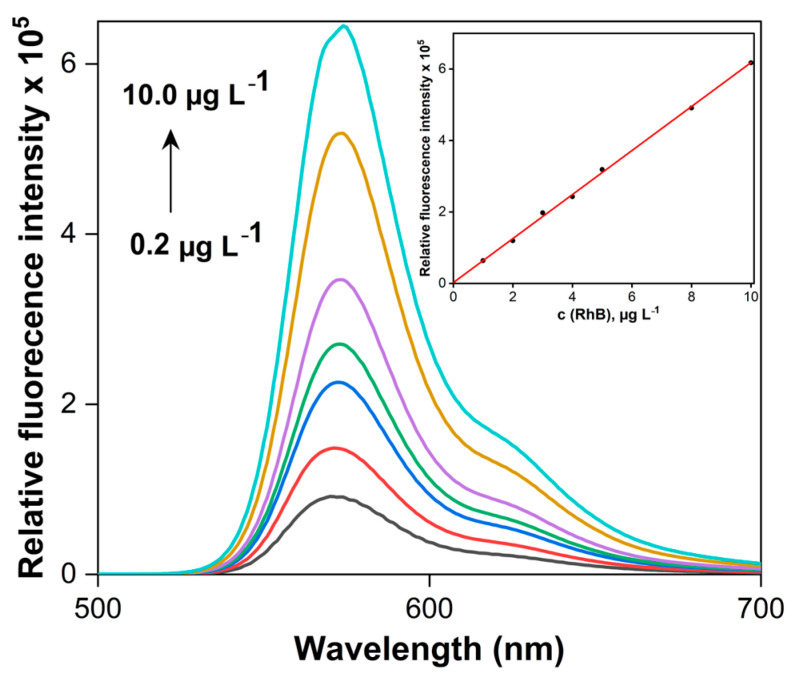
Fluorescence spectra and calibration curve at different RhB concentration. pH = 3.0; TBAB:hexanol (1:3) = 100 µL.

With increasing RhB concentration, the fluorescence intensity of DES extracts composed of TBAB:hexanol in the ratio 1:3 significantly increases (Figure 3). When microextraction was performed into 100 µL DES within the RhB concentration range from 0.2 to 10.0 µg L^−1^, the calibration curve was discovered to be linear. The regression equation, where y represents the relative fluorescence intensity and x is the concentration of RhB in µg L^−1^, had the form y = 61,590x + 2391. The analytical parameters of the VALLME-FLD method are summarized in Table 1. To characterize the efficiency of the microextraction procedure, the preconcentration factor (PF), defined as the ratio of the volumes of the aqueous and organic phases, and the enrichment factor (EF), which is calculated based on the ratio of the volumes of extracts without concentration and using 100 µL DES, are used. PF corresponds to a value of 50, and the EF value is 58.

To compare the analytical characteristics and prove the advantages of using the DES-VALLME-FLD method, the authors created a calibrated curve of RhB after microextraction using a DES composed of TBAB:hexanol (1:3), followed by UV–Vis absorbance measurement (DES-VALLME-UV–Vis). The linearity of the calibration curve in the case of microextraction into 100 µL DES was observed in the RhB concentration range from 0.0025 to 1.0 mg L^−1^. The regression equation of the straight line was y = 1.1078x + 0.0181 (y represents absorbance, x represents concentration of RhB in mg L^−1^), with a correlation coefficient of R^2^ = 0.9976. The LOD value was 0.75 µg L^−1^, and the LOQ value was 2.5 µg L^−1^. Based on these results, it can be said that the LOD and LOQ values when using the microextraction method with fluorescence detection are more than an order of magnitude lower than in the case of the analogous method with UV–Vis detection of microextracts. The analytical parameters of the VALLME-FLD and VALLME-UV–Vis methods are summarized in Table 1.

**Figure 4:**
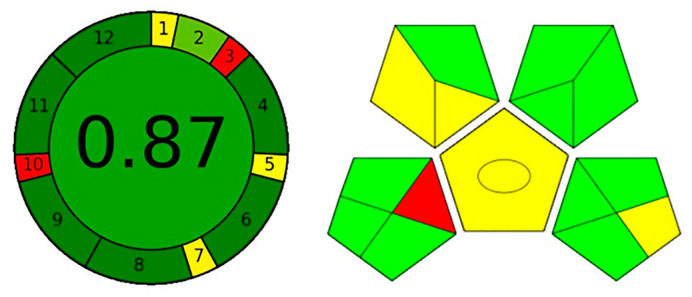
Assessment of the greenness of the proposed method using AGREE and GAPI.

The environmental friendliness of the DES-VALLME-FLD method was assessed using green evaluation metrics such as Analytical GREEnness (AGREE) and Green Analytical Procedure Index (GAPI). AGREE using GUI software evaluates the method according to 12 parameters that relate to the 12 principles of green analytical chemistry, and our method achieved a score of 0.87. GAPI assesses the environmental friendliness of the entire analytical methodology, from sampling to final determination. The green color of the individual pictogram is associated with a low environmental impact, yellow refers to a medium environmental impact, and red means a high environmental impact. The results of the greenness analyses performed using the GAPI technique for the VALLME-FLD method are shown in Figure 4.

By performing five microextractions of spiked samples at two levels of RhB concentrations (3.0 and 5.0 μg L^−1^) for two consecutive days, the accuracy and correctness of the proposed method were verified. The results are shown in Table 2. Satisfactory yield data ranging from 94.6 to 103.7% were obtained, with a relative standard deviation of 2.9 to 4.1%.

### 2.5. Theoretical Calculations

Theoretical calculations were used to study the dye molecule solvation at the molecular level to better understand the experimental extraction process. Considering the polyfunctional nature of the RhB molecules, the first step in modeling such molecules’ properties should be the determination of correct structural parameters. Moreover, as the RhB molecule is pH-sensitive and can have different structures in different pH mediums, it was crucial to select a proper structural form during the modeling. Only in such a case is it possible to obtain interpretable results that will explain and reproduce the experimentally observed peculiarities. In the first step, the pKa values of microspecies of RhB at different pH levels were estimated using the program Chemicalize. A few principally different structures were selected as input structures for the calculations. Monocationic cyclic forms containing a lactone moiety are the predominant forms at a pH of about 4.3 (Figure S1). RhB^+^ forms, with the localization of a positive charge over the diethylamino group nitrogen, are most stable at a pH of about 4.3 (Figure S2). Finally, the monocationic forms, with the localization of a positive charge over the xanthylium oxygen atom, were calculated to be predominant at a pH of about 2.9 (Figure S3). It should be highlighted that these results are preliminary and aimed only to roughly estimate the existence of microstates at various pH levels. The accuracy of the pKa values calculated by Chemicalize is characterized by a root mean square error (RMSE) of 1.11 and a Pearson correlation coefficient of 0.88 [52,53]. For a deeper understanding of the relative stability of the forms with different charges, density functional theory (DFT) calculations were performed.

**Scheme 1:**
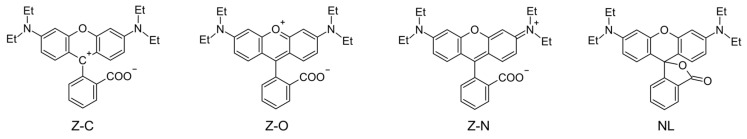
Three non-charged zwitterionic structures, Z-C, Z-O, Z-N, and the cyclic lactone form NL.

Both experimental and theoretical data show that the neutral, monocationic, and dicationic forms co-exist in a relatively narrow pH range, around 2.0. That is why it is reasonable to compare the microsolvation species for all three forms. Scheme 1 presents neutral forms of RhB, namely, the three zwitterionic structures (Z-C, Z-O, Z-N) of the noncyclic form NL. It should be mentioned that the resonance formulas Z-C, Z-O, and Z-N represent the same structure; however, they show that the positive charge can localize over different atoms in the dye molecule.

**Scheme 2:**
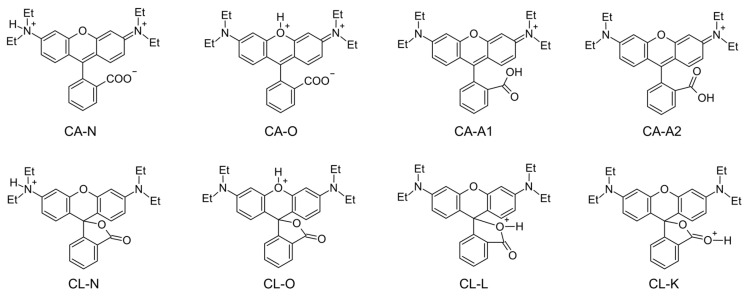
Mono-charged structures of RhB.

Figure S4 presents plots of Gibbs free energy of the neutral solvated RhB forms in media with different dielectric constants (Figure S4a,b). Upon the increase of the medium polarity (dielectric constant), the stability of the zwitterionic form increases. The zwitterionic form becomes predominant if the medium dielectric constant is higher than 10. The eight monocationic structures of RhB were obtained by protonating the non-charged forms (Scheme 2). The relative Gibbs free energy plots of the species at different values of the dielectric constant are presented in Figure S4. It should be mentioned that during the geometry optimization procedure, the authors were unable to obtain stable structures corresponding to formulas CA-N, CA-O, CL-L, and CL-K, as these structures were converted into CL-N, CL-O, CA-A1, and CA-A2, respectively; that is why CA-N, CA-O, CL-L, and CL-K were excluded from further analysis. At various polarities, the two predominant structures are CA-A1 and CA-A2, with the latter having a slightly higher stability than the former. The lactone forms CL-N and CL-O are significantly less preferable.

**Scheme 3:**
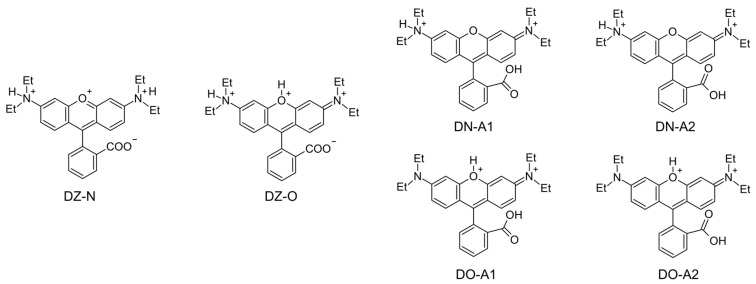
Dicationic structures of RhB.

Further protonation leads to dicationic species (see Scheme 3). Six structures were considered in total. Zwitterionic structures DZ-N and DZ-O are significantly more stable than the other forms and have very close energetic parameters. The relative Gibbs free energies of the neutral, monocationic, and dicationic species in water solution (the dielectric constant equals 80) are summarized in Table 3. From the table, it is clear that in a water medium, the zwitterionic neutral form is more stable than the lactone form, which makes structures Z-N, Z-O, and Z-C the predominant neutral forms. In the case of monocationic species, CA-A1 is the predominant form. Analogue DN-A2 structure is the predominant one in the series of dicationic species.

**Figure 5:**
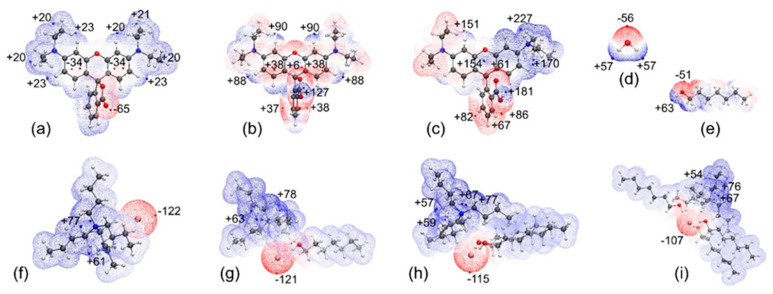
ESP isosurfaces of Z-N neutral form (**a**); CA-A2 cationic structure (**b**); DN-A2 dicationic structure (**c**); water (**d**); hexanol (**e**); TBAB (**f**); DES1 (**g**); DES2 (**h**); DES3 (**i**). Areas with positive and negative values of ESP are colored in blue and red, respectively.

The next step in understanding the microsolvation of RhB was to analyze the species’ molecular electrostatic potential (ESP). Analysis of ESP is a powerful approach to gain insight into reactivity and intra- and intermolecular interactions [54,55]. Figure 5 represents ESP isosurfaces, and the blue, white, and red areas correspond to maximal, medium, and minimal ESP values, respectively. The numerical values correspond to the extreme values of ESP in kcal/mol. The ESP minimum (–65 kcal/mol) of the neutral form Z-N corresponds to the carboxylic group (Figure 5a), which is consistent with the monoprotonated cationic form CA-A2, in which the proton is attached exactly to the carboxylic group. In the case of the CA-A2 cation (Figure 5b), all ESP values are positive. An ESP minimum of +6 kcal/mol is located over the central pyran ring; however, minima (red areas) are very delocalized over the whole molecule, which explains the failure to unequivocally predict the following protonation site, which should be one of the nitrogen atoms. A maximum of ESP corresponds to the proton of the carboxylic group and has a value of +127 kcal/mol; this shows that the interaction of CA-A2 with negatively charged sites X^−δ^ of other species will be realized through the formation of O–H···X hydrogen bonds. Similarly, ESP is positive in the DN-A2 dication, and the minima of +61 and +67 kcal/mol correspond to the ketone and hydroxyl oxygen atoms of the carboxylic group, respectively. Determining the most probable protonation sites in DN-A2 is not crucial, as further protonation of the dicationic form is outside the scope of the present study. Nevertheless, determining the ESP maxima of +227 and +181 kcal/mol located near the protons of protonated diethylamino and carboxylic groups is essential for defining these regions as possibly coordinated to negatively charged sites of other molecules.

Next, the authors focused on the ESP of the main solvents in the considered systems (Figure 5d–f). Water, 1-hexanol, and TBAB molecules are characterized as highly polar compounds. All these molecules have areas that can be considered as electron donors (low values of ESP) and acceptors (high values of ESP). The ESP minima correspond to oxygen atoms and to the bromide anion and are equal to –56 (water), –51 (1-hexanol), and –122 (TBAB) kcal/mol. Conversely, in the case of water, 1-hexanol, and TBAB, the ESP maxima correspond to hydrogen atoms and are equal to +57, +63, and +77 kcal/mol, respectively. Three clusters of TBAB:hexanol with component ratios of 1:1 (DES1), 1:2 (DES2), and 1:3 (DES3) were considered as model DESs. The association of TBAB and 1-hexanol into structural clusters of DESs does not influence ESP maxima; however, ESP was insignificantly lowered from –121 (DES1) to –115 (DES2) and to –107 (DES3) kcal/mol (Figure 5g–i). In all the considered clusters of TBAB and 1-hexanol, the hydrogen atoms of the hydroxyl group are coordinated to the bromide anion; this coordination leads to a slight charge transfer from bromide to the hydrogen atoms, which, in turn, slightly decreases the ESP near the bromide atoms.

**Figure 6:**
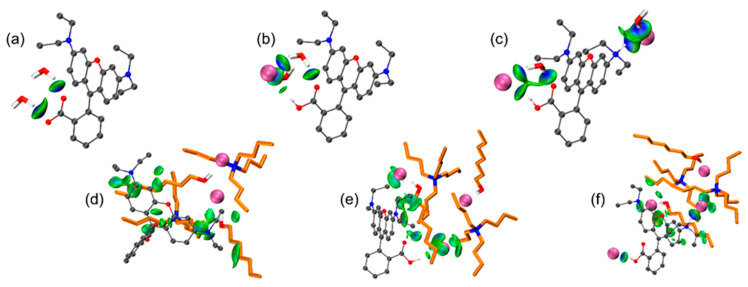
IGM isosurfaces of Z-N neutral form with two water molecules (**a**); CA-A2 bromide with two water molecules (**b**); DN-A2 dibromide with two water molecules (**c**); Z-N neutral form with two DES1 structures (**d**); CA-A2 bromide with two DES1 structures (**e**); DN-A2 dibromide with two DES1 structures (**f**). Ball-and-sticks representation was used for the dye. Water and DESs are shown as thick sticks. For clarity, only polar hydrogens are shown. H atoms are white; C atoms are black in the dyes and orange in DESs; N atoms are blue; O atoms are red; Br anions are pink. Areas of IGM colored in green and blue correspond to weak interactions and hydrogen bonds, respectively.

Further analysis of non-covalent intermolecular interactions in microsolvated forms of RhB was performed via independent gradient model (IGM) [56]. This analysis utilizes molecular geometry and promolecular density as necessary inputs, which makes it especially attractive for large systems. As microsolvated forms of RhB, the authors have considered six clusters of Z-N, CA-A2, and DN-N2 structures, with either two molecules of water or two molecules of DES1. It should be mentioned that the CA-A2 and DN-N2 cations were neutralized by adding one or two bromide anions, respectively; the bromides were added near the ESP maxima, and the structures were reoptimized. The clusters were generated by the rigid molecule artificial bee colony optimization method using the ABCluster 3.3 software [57,58]. The clusters with the intermolecular IGM isosurfaces are presented in Figure 6.

The blue areas in water-containing clusters correspond to strong hydrogen bonding (Figure 6a–c). Clusters with DES1 contain fewer blue areas, and most of the isosurfaces are green, which corresponds to weak dispersion attraction (Figure 6d–f). While the water molecules form strong hydrogen bonds only with the polar groups of the RhB forms, such as carboxyl and carboxylate groups, or bromide anions, the alkyl chains of DES1 are attracted to the different sites of the dye. That is why, using this visual approach, it is hard to distinguish the strength of interactions of the RhB forms with different solvents. The authors have tried to calculate the interaction energy between the RhB forms and the water/DES1 solvent. Considering the size of the systems with DES1 (219 atoms in Z-N(DES1)_2_, 221 atoms in CA-A2(DES1)_2_, and 223 atoms in DN-A2(DES1)_2_), the interaction energy was calculated using the PM7 semiempirical method [59], since the usage of high-accuracy ab initio methods for such large systems would be much more computationally expensive. The interaction energy between water molecules and RhB forms for clusters Z-N(H_2_O)_2_, CA-A2(H_2_O)_2_, and DN-A2(H_2_O)_2_ was found to be equal to –13.0, –8.8, and –11.0 kcal/mol, respectively. In the case of DES1, the solvent–solute interaction energy was found to be equal to –30.4, –10.0, and –18.4 kcal/mol for Z-N, CA-A2, and DN-A2 forms, respectively. According to the PM7 energies, all forms of RhB interact more strongly with DES1 than with water and, thus, are expected to be more soluble in DES1 and more easily extracted by DES1. Nevertheless, the selected models are limited in the number of selected solvent molecules; the addition of more solvent molecules will tremendously increase the calculation time, whereas the possible selection of less costly methods like molecular mechanics with force fields can significantly decrease the accuracy for such specific systems.

### 2.6. Hansen Solubility

That is why, in order to explain the experimental selective extraction of RhB with DESs, the authors decided to describe the system in terms of Hansen solubility parameters (HSPs) [60]. Based on the principle that like dissolves like, HSPs become powerful solubility descriptors that allow one to estimate the solubility of diverse compounds in various solvents and mixtures [61,62,63]. Whereas HSPs are known for common solvents, the parameterization of new compounds is quite a tedious process that requires numerous experimental trials [60]. However, the authors propose a relatively simple procedure for the determination of HSPs based on theoretical semiempirical calculations that can be performed in minutes on a common laptop.

The HSP model was developed based on the Hildebrand solubility parameter (δ), which Hansen had proposed breaking down into dispersion (δ_D_), polarity (δ_P_), and hydrogen bond (δ_H_) components that should more accurately describe intermolecular interactions [60]. Equation (1) describes the relationship between the above-mentioned parameters.

δ^2^ = δ_D_^2^ + δ_P_^2^ + δ_H_^2^ (1)

Usually, δ_D_, δ_P_, and δ_H_ HSPs are considered as axes of tridimensional plots, and solute–solvent miscibility is estimated based on the distance between solute and solvent in this 3D space. The distance between the solute (s) and the solvent (sol) is denoted R_a_ and calculated according to Equation (2).

R_a_^2^ = 4 × (δ_D_^s^ − δ_D_^sol^)^2^ + (δ_P_^s^ − δ_P_^sol^)^2^ + (δ_H_^s^ − δ_H_^sol^)^2^ (2)

To determine if the solute and solvent parameters are close enough for good solubility, a relative energy difference (RED) parameter is considered. RED can be calculated by the division of R_a_ by the so-called interaction radius R_0_, which is specific for a solute molecule under study. Good solvents are enclosed inside a sphere of diameter R_0_, often called a Hansen sphere, which means that RED < 1. Bad solvents form immiscible solute-solvent systems and are characterized by RED > 1. In the case when RED equals 1, the solute is only partly soluble. The R_0_ value of RhB forms was selected to be equal to 13, which allowed us to distinguish the solubility in different solvents.

Six solvents, amyl acetate, chloroform, octanol, tetrachloromethane, toluene, and water, were used in this work as a small training set. The PM7 semiempirical Hamiltonian [59] was used for the geometry relaxation and calculation of COSMO [64] molecular volume (V_COSMO_), dipole moment (µ), polarizability (α), and Mulliken partial charges (Q). These data were used to calculate dispersion (P_D_), polarity (P_P_), and hydrogen bond (P_H_) parameters according to Equations (3)–(5).

(3) $$P_{D}=\frac{\alpha^{8/9}}{V_{MOL}}$$

(4) $$P_{P}=37.4\frac{\mu }{\sqrt{V_{MOL}}}$$

(5) $$P_{H}=1000\frac{\max\left[Q\left(H\right)\right]}{\sqrt{V_{MOL}}}$$

**Figure 7:**
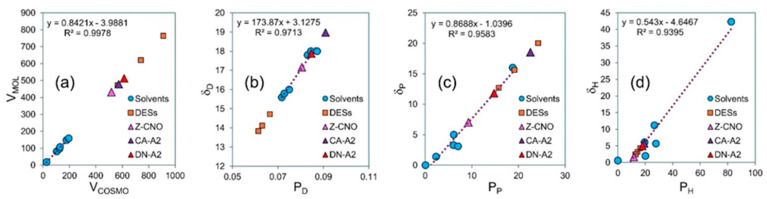
Correlations between molecular volume V_MOL_ and calculated V_COSMO_ (**a**); P_D_ and δ_D_ (**b**); P_P_ and δ_P_ (**c**); and P_H_ and δ_H_ (**d**). The dashed lines correspond to the linear equations on the graphs.

Equation (4) was described by Hansen [60]. Equation (3) was found empirically, knowing that the strength of dispersion interactions is related to the polarizability of the molecules. Equation (5) was proposed based on the idea that the information on the ability to form hydrogen bonds is hidden in the partial charges of hydrogen atoms. The maximal charge is expected for compounds containing more polar -X–H bonds and, thus, will form stronger -X–H···X hydrogen bonds. The square root of molecular volume V_MOL_ was adopted similarly to known Equation 4, and a coefficient of 1000 was used to achieve higher values, which are easier to work with. Moreover, V_m_ can be calculated for common liquids, knowing density and molecular weight; however, when the density is not known, V_MOL_ can be found from V_COSMO_, as a very good correlation was found (see Figure 7). Also, good correlations were found between proposed parameters P_D/P/H_ and HSPs δ_D/P/H_ (see Figure 7). The corresponding linear equations and correlation parameters are presented in Figure 7. These linear equations were used for the prediction of the HSPs of DES1 through DES3 and the forms of RhB. Considering that HSPs are mostly used for non-charged species, the monocationic (CA-A2) and dicationic (DN-A2) forms of RhB were neutralized by adding to their structures one and two bromide anions, respectively. The anions were initially located close to the points corresponding to ESP maxima, and the resulting structures were optimized by the PM7 method.

**Figure 8:**
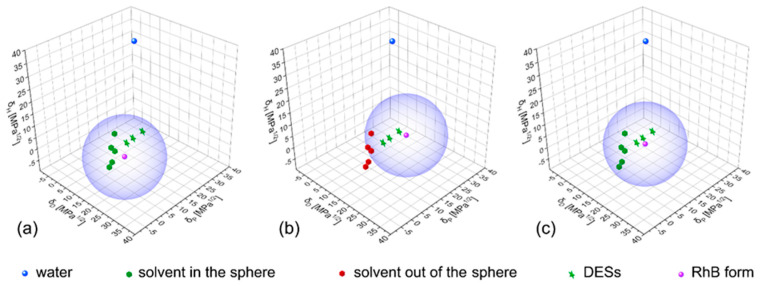
Hansen’s spheres determined for Z-N (**a**), CA-A2 + Br^−^ (**b**), and DN-A2 + 2Br^−^ (**c**), calculated according to Equations (2)–(5) with the MOPAC 22.1.1 program [66].

Hansen’s solubility spheres for Z-N, CA-A2 + Br^−^, and DN-A2 + 2Br^−^ structures are presented in Figure 8. The RhB forms are presented as purple dots in the center of the translucent purple Hansen’s sphere. All the considered DESs (presented as green stars) are in the spheres, which means that they are good solvents for the RhB forms. In the case of CA-A2 + Br^−^, other considered solvents (amyl acetate, chloroform, octanol, tetrachloromethane, and toluene) are out of the sphere of the selected R_0_ of 13, which makes these solvents poorer solvents, or even bad solvents for the CA-A2 form of RhB; it is interesting to notice that the neutral and dicationic forms should be more soluble in these common solvents. For all forms of RhB, water (blue dot in Figure 8) is too far from the sphere, which indicates that it is a bad solvent. Indeed, considering the solubility of RhB chloride is ~15 g/L (~0.031 mol/L), RhB can be characterized as poorly soluble [65].

### 2.7. Analytical Application

To demonstrate the practicality of the proposed procedure, a method was developed and applied to determine RhB in real samples (tap water, energy drink, and lipstick). The samples were spiked with different concentrations of RhB and analyzed five times according to the established procedure. The recovery data obtained were satisfactory, with values ranging from 95.8% to 104.2% and a relative standard deviation from 2.1% to 3.5%. The results are presented in Table 4.

### 2.8. Comparison with Other Methods

In order to define the analytical properties of the DES-VALLME-FLD method and compare its analytical characteristics with previous studies in the literature, the validation parameters of the analytical method for RhB microextraction were determined. Comparative characteristics of some current UV–Vis and fluorescence methods for determining RhB are presented in Table 5.

## 3. Materials and Methods

### 3.1. Materials

All chemicals employed in the experiment were of analytical-grade purity and were dissolved in distilled water. A RhB stock solution (Merck, Darmstadt, Germany), having a concentration of 1 × 10^−3^ M, was prepared by dissolving 0.0480 g of RhB in water and diluting to a final volume of 100 mL. Working solutions of RhB with concentrations ranging from 1 × 10^−4^ to 1 × 10^−7^ M were prepared by diluting the stock solution with double distilled water to the desired concentrations. DES extraction mixtures were prepared using tetrabutylammonium bromide (Merck, Germany) and alcohol (hexanol, octanol, and decanol) (Merck, Germany) in different molar ratios. Tetrachloromethane was purchased from Merck (Germany), n-amyl acetate and chloroform from Centralchem (Bratislava, Slovakia), and toluene from ITES (Vranov nad Topľou, Slovakia). The pH was adjusted using HCl (ITES, Slovakia), NaOH (Centralchem, Slovakia), and a buffer solution mixing 1 M ammonium hydroxide (ITES, Slovakia) and 1 M acetic acid (ITES, Slovakia).

#### Applied Instruments

Shimadzu (Kyoto City, Japan) RF-6000 luminescence spectrophotometer was used for fluorescence measurements. The measurements utilized a High-Precision Cell fluorescence microcuvette (3 × 3 mm) from Hellma Analytics (Müllheim, Germany) and a compatible ultra-micro cell holder from Shimadzu. A Specord S600 spectrophotometer from Analytic Jena (Jena, Germany) equipped with a 1 cm quartz microcuvette was used for spectrophotometric measurements. Hellma Analytics standard optical probe, connected with the spectrophotometer, was applied for in situ measurements. 70 Vio pHmetro portable potentiometer (XS Instruments, Carpi, Italy) was used for pH measurements. A Fisherbrand Vortex Stirrer 3000 (Fisher Scientific, Hampton, VA, USA) facilitated mixing, while phase separation of the organic and aqueous phases was achieved using a MRC BLCEN-208 centrifuge from LabGear (Washington, DC, USA). DES extraction mixtures were prepared using an AREX DIGITAL Heating Magnetic stirrer from VELP SCIENTIFICA (Usmate Velate, Italy).

### 3.2. Methods

#### 3.2.1. Preparation of DESs

DES extraction systems were prepared using TBAB and an alcohol solvent (hexanol, octanol, and decanol) in molar ratios of 1:1; 1:2, and 1:3. Using an electric stirrer, all prepared DES mixtures were dissolved at 90 °C and 300 rpm until homogeneous, clear solvent systems were formed. The DES extraction mixtures were allowed to cool after complete dissolution. After cooling, the solvents were used.

#### 3.2.2. Characterization of DESs by FTIR Technique

The FTIR spectra of the prepared materials were measured and recorded at laboratory temperature with a Nicolet Avatar FTIR 6700 spectrometer (SpectraLab Scientific, Markham, Canada) in the wavenumber range of 4000–400 cm^−1^. Transmission FTIR measurements were performed using the ATR technique using ATR Smart Orbit^TM^ (SpectraLab Scientific, Markham, Canada). 

FTIR spectra of hexanol, TBAB, and DESs composed of TBAB and hexanol in molar ratios of 1:1, 1:2, and 1:3 are presented in Figure S5. The assignment of IR peaks to specific vibrations was performed through comparison with the literature data [67] and by the vibrational mode automatic relevance determination using vibAnalysis 1.2.2 [68]. The FTIR peaks’ assignments for hexanol, TBAB, and DESs composed of TBAB:hexanol in molar ratios of 1:1, 1:2, and 1:3 are presented in Tables S1–S5. In general, FTIR spectra of DESs are similar to the spectra of hexanol, with a few differences caused by the presence of TBAB. Particularly, the differences in O–H stretching vibrations should be highlighted. Thus, in the case of DESs with TBAB:hexanol ratios equaling 1:1, 1:2, and 1:3, the peaks corresponding to O–H stretching are redshifted to 3342, 3348, and 3354 cm^−1^, respectively. The O–H stretching of pure hexanol corresponds to the peak at 3325 cm^−1^. Moreover, in the case of hexanol, the bending of the H_2_C–O–H group corresponds to the peak at 1119 cm^−1^, whereas in the case of DESs, this bending appears at 1115 cm^−1^. These shifts can be explained by the coordination of OH groups to the bromide anion and the formation of strong O–H···Br^−^ interactions. 

#### 3.2.3. Sample Preparation

A 50 mg sample of the lipstick (dm-drogerie markt) was accurately weighed and dissolved in 50 mL of distilled water at 60 °C. After cooling, undissolved particles were removed using a paper filter (blue ribbon). Subsequently, a 1 mL aliquot was used for analysis.

The energy drink (Redbull) was sonicated using an ultrasonic water bath for 5 min to remove CO_2_ bubbles. Subsequently, a 1 mL aliquot was used for analysis.

#### 3.2.4. Procedure for the Determination of RhB with DES-VALLME-FLD

RhB was added to 15 mL plastic centrifuge tubes in volumes ranging from 0.1 to 1.0 mL, in order to achieve concentrations ranging from 0.2–10.0 µg L^−1^. Following this, pH 3.0 buffer solution was added, and the mixture was then diluted with 5 mL of distilled water. Subsequently, 100 µL of DES (TBAB:hexanol in molar ratio 1:3) was added to the test tubes. The system containing two immiscible phases was then subjected to vortex mixing for 15 s at 2000 rpm. The resulting turbid emulsion underwent centrifugation for 3 min at 1500 rpm. Afterward, the DES extraction phase was collected using a micropipette and transferred to a fluorescent microcuvette. Fluorescence measurements were conducted at 574 nm, with excitation at 557 nm. The obtained results were evaluated using a calibration curve.

#### 3.2.5. Theoretical Methods and Software

The starting geometries of cyclic lactone and non-cyclic forms of RhB were constructed based on X-ray geometries deposited in the Cambridge Crystallographic Data Center (CCDC) under IDs 152610 [69] and 991037 [70], respectively. For consistency, the same conformation of the diethylamino group was selected for all the considered structures, and it was selected to be equal to that of the CCDC ID 991037 structure.

Geometry optimization was performed using density functional theory (DFT) at the B3LYP/def2-SVP level of theory [71,72]. The larger def2-TZVPP basis set was utilized for calculating the total electronic energies [72]. In the case of DFT calculations, the solvent effects, which often play a crucial role on the properties of molecules [73], were included using the conductor-like polarizable continuum model (CPCM) [74]. DFT calculations were done with the ORCA program [75]. The MOPAC program was used for semiempirical calculations with the PM7 Hamiltonian [59,65] and the COSMO solvation model [66]. Analysis of electrostatic potential (ESP) and weak interactions by the independent gradient model (IGM) was performed with the Multiwfn code [56,76,77]. Molecular clusters were generated according to the artificial bee colony algorithm, which was implemented in the ABCluster 3.3 program [57,58]. Visualization was performed with the VMD 1.9.4 software [78].

## 4. Conclusions

In this work, a new green method for the determination of rhodamine B (RhB) by deep eutectic solvent-based vortex-assisted liquid–liquid microextraction with fluorescence detection (DES-VALLME-FLD) was developed. The DES-VALLME-FLD method offers several advantages for analysis:

(a) The method is characterized by high sensitivity; the calculated LOD values (0.023 µg L^−1^) are lower than the characteristics of most known RhB determination methods.
(b) The DES-VALLME procedure is simple, characterized by a simple short extraction time and a high preconcentration and enrichment factor (EF value is 58 and PF value is 50).
(c) The correct choice of green DES eliminates the need to add conventional organic solvents and additional reagents for RhB extraction and determination.

DFT calculations allowed for determination of the most stable forms of RhB dye in solution and were used to study their microsolvation in DES in detail. Analysis of electrostatic potential allocated the most polar (positively/negatively charged) sites in the RhB forms and explained strong interaction with DES clusters. Independent gradient model analysis of intermolecular interactions testified to the formation of van der Waals attraction between the RhB forms and the alkyl chains of the DES clusters. According to semiempirical calculations, the interaction energy between non-charged/monocationic/dicationic RhB forms and DES was 1.2–27.4 kcal/mol higher compared to water.

A simplified computational approach to Hansen solubility parameters allowed the authors to calculate the Hansen solubility spheres for the dye forms and to estimate their solubility in three DESs. The obtained values characterize DESs as good solvents for the monocationic form of RhB, whereas the other considered solvents were characterized as bad for this particular RhB form.

The DES-VALLME-FLD method was successfully applied for the separation, preconcentration, and determination of RhB in food samples, cosmetic products, and water samples. The authors assume that such an innovation will also be useful for the determination of other analytes.

## Figures and Tables

**Table 1:** Analytical parameters of the VALLME-FLD and VALLME-UV–Vis methods.

	VALLME-FLD	VALLME-UV–Vis
DES (TBAB:hexanol (1:3)), µL	100	100
Linear range, µg L^−1^	0.2–10.0	2.5–1000
Calibration equation	y = 61,590x + 2391	y = 1.1078x + 0.0181
Correlation coefficient	0.9991	0.9976
LOD, µg L^−1^	0.023	0.75
LOQ, µg L^−1^	0.169	2.5
Enrichment factor	58	25
Preconcentration factor	50	50

**Table 2:** Precision and accuracy data for the determination of RhB in model samples (*n* = 5, *p* = 0.95).

RhB Added, µg L^−1^	Intra-Day			Inter-Day		
	RhB Determined, µg L^−1^	RSD, %	Recovery, %	RhB Determined, µg L^−1^	RSD, %	Recovery, %
3.0	2.87 ± 0.12	3.3	95.7	3.11 ± 0.16	4.1	103.7
5.0	4.73 ± 0.17	2.9	94.6	4.99 ± 0.25	4.0	99.8

**Table 3:** The relative Gibbs free energies (G, in kcal/mol) and mole fractions (B, in%) of the neutral, monocationic, and dicationic species in a water solution. The dielectric constant is 80, and the pH influence is neglected.

	Neutral		Monocationic				Dicationic					
Structure	Z-N	NL	CA-A1	CA-A2	CL-N	CL-O	DN-A1	DN-A2	DO-A1	DO-A2	DZ-N	DZ-O
G	0.0	2.5	1.0	0.0	15.5	52.2	1.1	0.0	37.2	36.2	10.7	49.9
B	98.6	1.4	15.6	84.4	0.0	0.0	13.5	86.5	0.0	0.0	0.0	0.0

**Table 4:** Determination of RhB in real samples (*n* = 5, *p* = 0.95).

	RhB Concentration, µg L^−1^			
Samples	Added	Found	RSD, %	Recovery, %
Tap water	-	≤LOQ	-	-
	3.0	2.91 ± 0.09	2.6	96.9
	5.0	5.20 ± 0.23	3.5	103.9
Lipstick	-	≤LOQ	-	-
	3.0	3.13 ± 0.12	3.0	104.2
	5.0	5.12 ± 0.15	2.4	102.3
Energy drink	-	≤LOQ	-	-
	3.0	2.87 ± 0.07	2.1	95.8
	5.0	4.89 ± 0.17	2.8	97.9

**Table 5:** Comparative characteristics of the developed method with the UV–Vis and fluorescence methods proposed in the literature for the RhB determination.

Separation/Preconcentration Procedures	Detection Technique	Linear Range (µg L^−1^)	R^2^	LOD (µg L^−1^)	RSD (%)	Recovery (%)	Reference
Solid-phase extraction based on the adsorption on the resin and elution in a mini chromatographic column	UV–Vis	250.0–3000	0.9996	3.14	5.0	96.0–118.0	[10]
Simultaneous liquid-phase microextraction using a deep eutectic solvent	UV–Vis	42.0–3000	0.9992	13.0	7.0	96.0–101.0	[11]
Ultrasonic liquid-phase microextraction using a deep eutectic solvent	UV–Vis	29.0–400.0	0.9970	8.0	3.6	90.0–108.0	[12]
Cloud point extraction with a surfactant	FLD	0.047–100.0	0.9990	0.01	-	95.2–104.8	[16]
On-line surfactant mediated extraction using a glass wool mini-column to collect the bile salt coacervate phase	FLD	0.4–479.0	0.9933	0.12	-	95.3–118.9	[17]
Solid–phase extraction by ionic liquid coated magnetic nanoparticles	FLD	0.40–140.0	0.9993	0.06	0.45	97.0–103.5	[18]
Magnetic solid-phase extraction based on a 3D reduced graphene oxide composite	FLD	0.025–150.0	0.9991	0.01	0.5–2.0	95.7–99.9	[19]
Molecular imprinting solid-phase extraction using novel magnetic molecularly imprinted polymers as dispersant sorbents	FLD	2.5–112.5	0.9934	0.45	0.9–5.4	94.9–102.5	[20]
Vortex-assisted liquid–liquid microextraction using a deep eutectic solvent combined	FLD	0.2–10.0	0.9991	0.02	2.1–3.5	95.8–104.2	This work

## Data Availability

Data are contained within the article and Supplementary Materials.

## Supplementary Materials

The following supporting information can be downloaded at: https://www.mdpi.com/article/10.3390/molecules29143397/s1, Figure S1: The distribution of microspecies of lactone (cyclic) forms of RhB at different pH. Calculated with Chemicalized.; Figure S2: The distribution of microspecies of non-cyclic forms of RhB with localization of a positive charge over the diethylamino group nitrogen atom at different pH. Calculated with Chemicalized; Figure S3: The distribution of microspecies of non-cyclic forms of RhB with localization of a positive charge over the pyran ring oxygen atom at different pH. Calculated with Chemicalized; Figure S4: Relative Gibbs free energies of solvated structures. Neutral zwitterionic forms (a) and the cyclic lactone form (b). Mono-cationic forms CA-A2 (c), CA-A1 (d), CL-N (g), CL-O (f). Di-cationic forms DN-A1 (g), DN-A2 (h), DO-A1 (i), DO-A2 (j), DZ-N (k), DZ-O (l); Figure S5: FT-IR spectra of hexanol (a), TBAB (e), and DESs composed of TBAB and hexanol in molar ratios of 1:1 (b), 1:2 (c), and 1:3 (d): (A) full spectra; (B) zoomed region of 2700–3600 cm^−1^; (C) zoomed region of 650–1600 cm^−1^; Table S1: Assignment of specific vibrations to the selected peaks in the FTIR spectrum of hexanol; Table S2: Assignment of specific vibrations to the selected peaks in the FTIR spectrum of DES with TBAB: hexanol ratio equals 1:1; Table S3: Assignment of specific vibrations to the selected peaks in the FTIR spectrum of DES with TBAB: hexanol ratio equals 1:2; Table S4: Assignment of specific vibrations to the selected peaks in the FTIR spectrum of DES with TBAB: hexanol ratio equals 1:3; Table S5: Assignment of specific vibrations to the selected peaks in the FTIR spectrum of TBAB.
