# Supplementary material for: An Innovative Vortex-Assisted Liquid-Liquid Microextraction Approach Using Deep Eutectic Solvent: Application for the Spectrofluorometric Determination of Rhodamine B in Water, Food and Cosmetic Samples
Source: Molecules. 2024 Jul 19;29(14):3397. doi: 10.3390/molecules29143397 (PMC11280433; doi:10.3390/molecules29143397)
Supplement: Supplementary file 1 [file molecules-29-03397-s001.zip › molecules-3103785-supplementary.pdf]

Supplementary

An Innovative Vortex-Assisted Liquid-Liquid Microextraction Approach Using Deep Eutectic Solvent: Application for the Spectrofluorometric Determination of Rhodamine B in Water, Food and Cosmetic Samples

Sofia Kakalejčíková [1], Yaroslav Bazeľ [1,*], Van Anh Le Thi [1] and Maksym Fizer [2]

1 Department of Analytical Chemistry, Institute of Chemistry, Pavol Jozef Šafárik University in Košice, 040 01 Košice, Slovakia; sofia.kakalejcikova@student.upjs.sk (S.K.); van.anh.le.thi@student.upjs.sk (V.A.L.T.)
2 Department of Chemistry, University of Nevada, Reno, 1664 N. Virginia Street, Reno, NV 89557-0216, USA; mmfizer@gmail.com
* Correspondence: yaroslav.bazel@upjs.sk

Supplementary information

CONTENT

Item	Page
**Figure S1.** The distribution of microspecies of lactone (cyclic) forms of RhB at different pH. Calculated with Chemicalized.	2
**Figure S2.** The distribution of microspecies of non-cyclic forms of RhB with localization of a positive charge over the diethylamino group nitrogen atom at different pH. Calculated with Chemicalized.	3
**Figure S3.** The distribution of microspecies of non-cyclic forms of RhB with localization of a positive charge over the pyran ring oxygen atom at different pH. Calculated with Chemicalized.	4
**Figure S4.** Relative Gibbs free energies of solvated structures. Neutral zwitterionic forms (a) and the cyclic lactone form (b). Mono-cationic forms CA-A2 (c), CA-A1 (d), CL-N (g), CL-O (f). Di-cationic forms DN-A1 (g), DN-A2 (h), DO-A1 (i), DO-A2 (j), DZ-N (k), DZ-O (l).	5
**Figure S5.** FT-IR spectra of hexanol (a), TBAB (e), and DESs composed of TBAB and hexanol in molar ratios of 1:1 (b), 1:2 (c), and 1:3 (d): (A) full spectra; (B) zoomed region of 2700–3600 cm^{-1}; (C) zoomed region of 650–1600 cm^{-1}.	6
**Table S1**. Assignment of specific vibrations to the selected peaks in the FTIR spectrum of hexanol.	7
**Table S2**. Assignment of specific vibrations to the selected peaks in the FTIR spectrum of DES with TBAB: hexanol ratio equals 1:1.	7
**Table S3**. Assignment of specific vibrations to the selected peaks in the FTIR spectrum of DES with TBAB: hexanol ratio equals 1:2.	8
**Table S4**. Assignment of specific vibrations to the selected peaks in the FTIR spectrum of DES with TBAB: hexanol ratio equals 1:3.	8
**Table S5**. Assignment of specific vibrations to the selected peaks in the FTIR spectrum of TBAB.	9

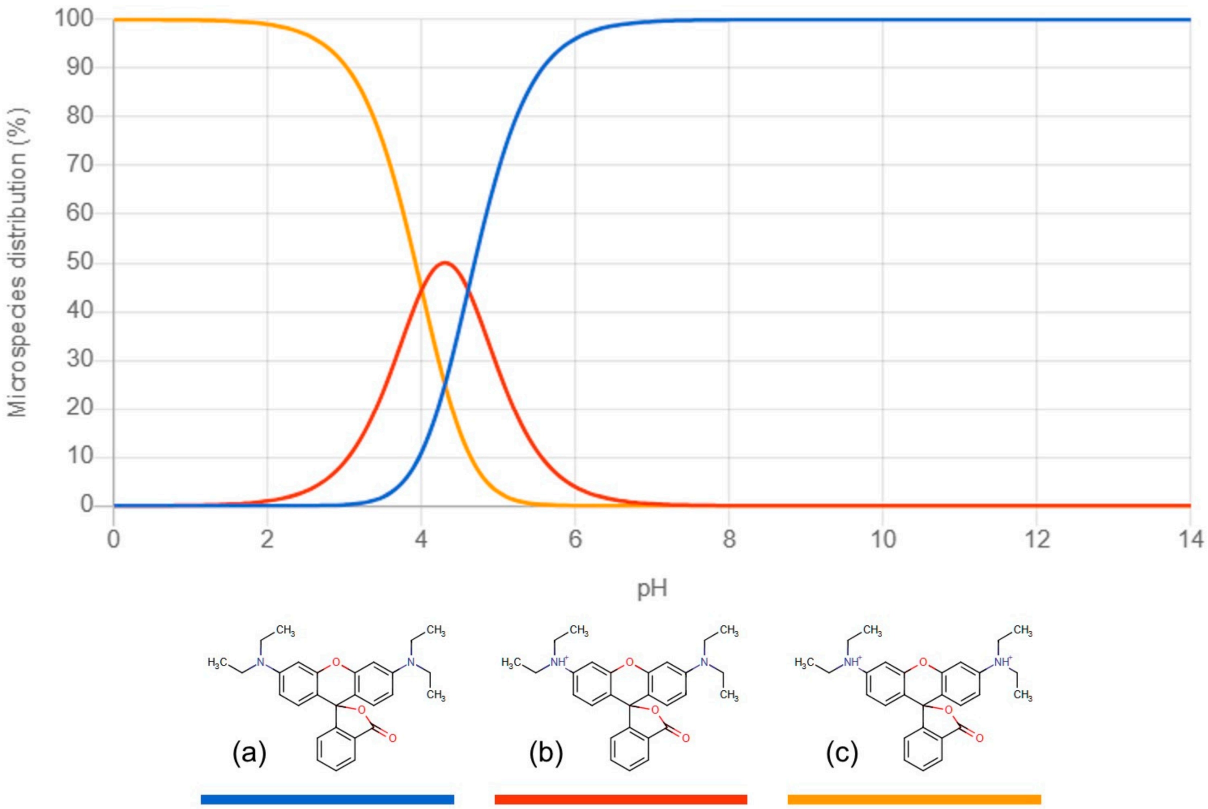

Figure S1. The distribution of microspecies of lactone (cyclic) forms of RhB at different pH. Calculated with Chemicalized.

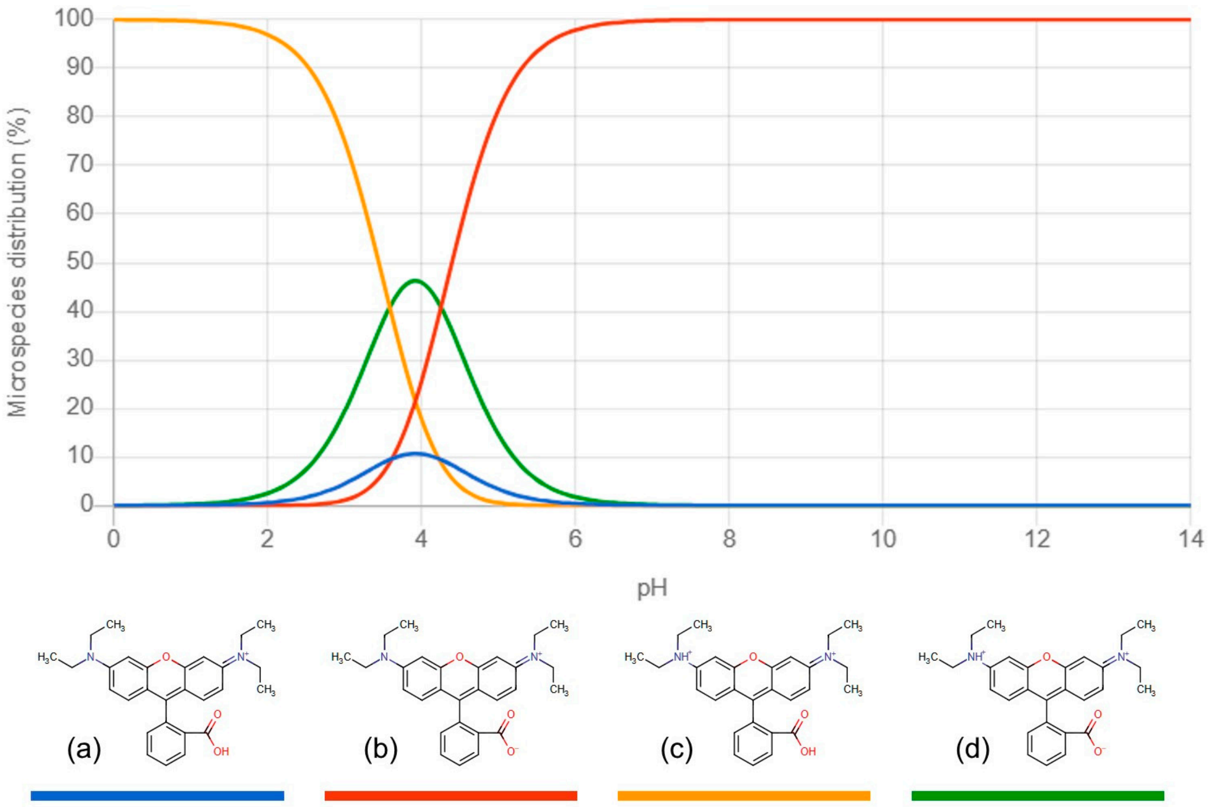

Figure S2. The distribution of microspecies of non-cyclic forms of RhB with localization of a positive charge over the diethylamino group nitrogen atom at different pH. Calculated with Chemicalized.

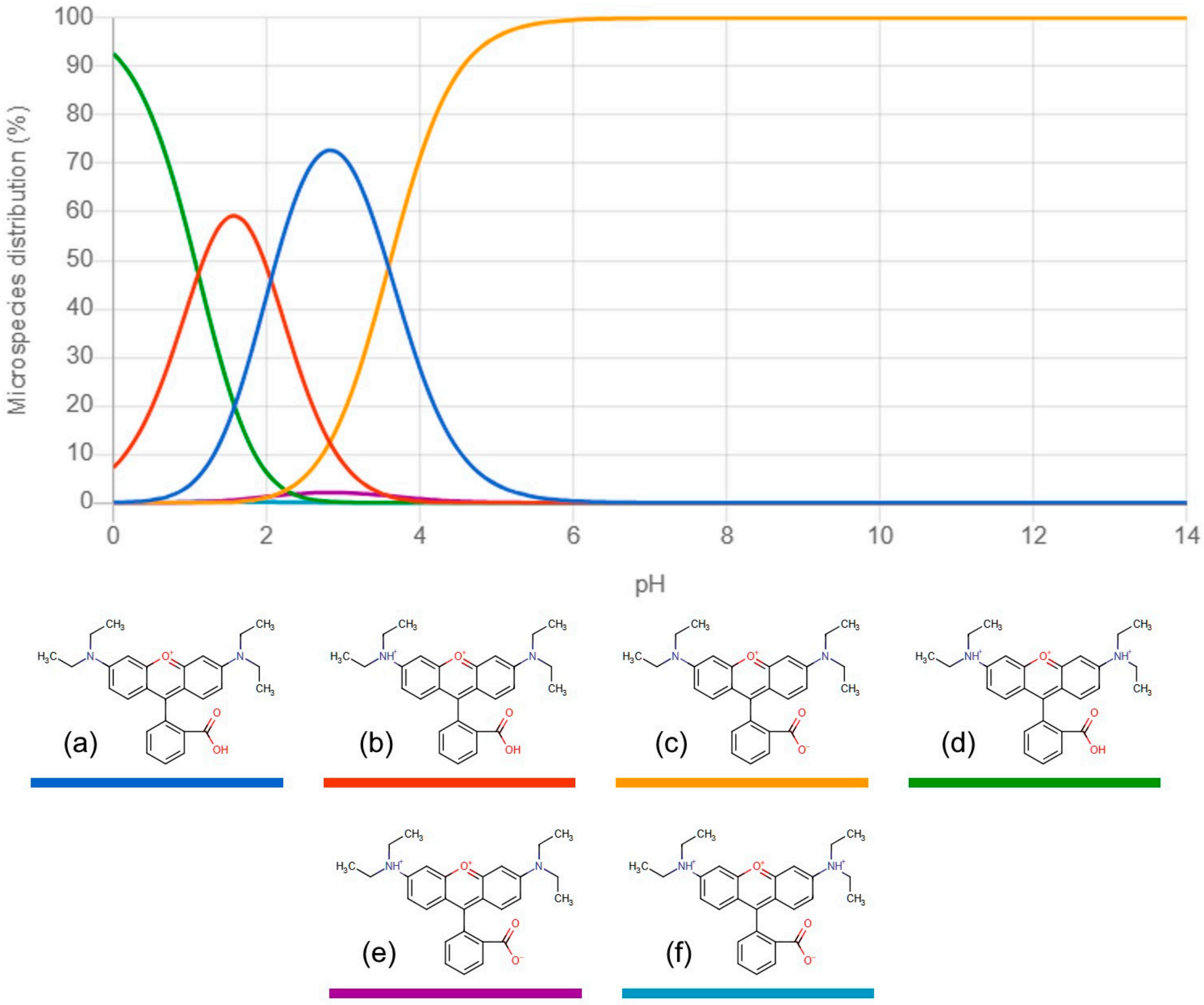

Figure S3. The distribution of microspecies of non-cyclic forms of RhB with localization of a positive charge over the pyran ring oxygen atom at different pH. Calculated with Chemicalized.

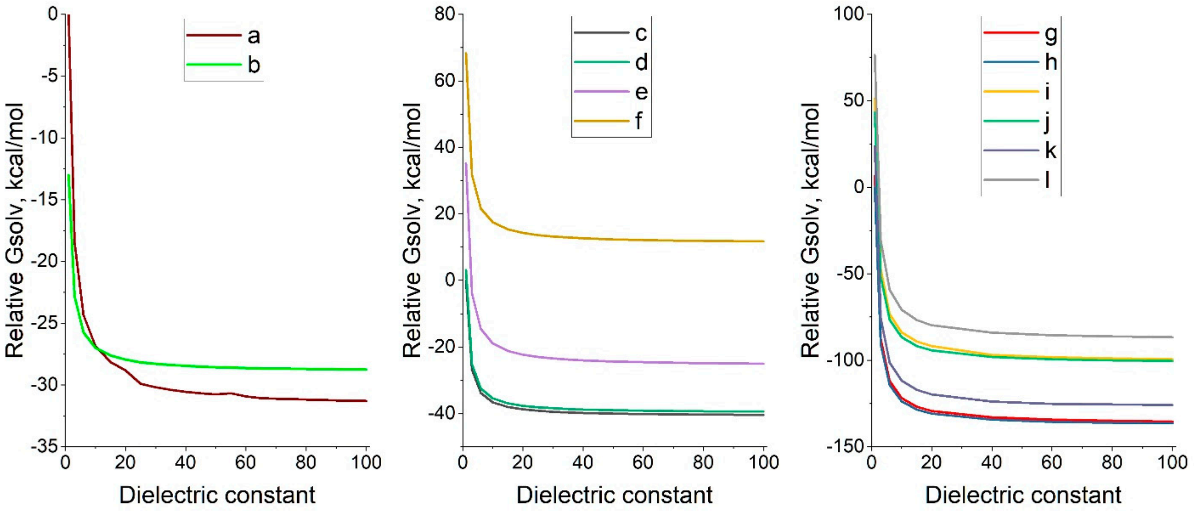

Figure S4. Relative Gibbs free energies of solvated structures. Neutral zwitterionic forms (a) and the cyclic lactone form (b). Mono-cationic forms CA-A2 (c), CA-A1 (d), CL-N (g), CL-O (f). Di-cationic forms DN-A1 (g), DN-A2 (h), DO-A1 (i), DO-A2 (j), DZ-N (k), DZ-O (l).

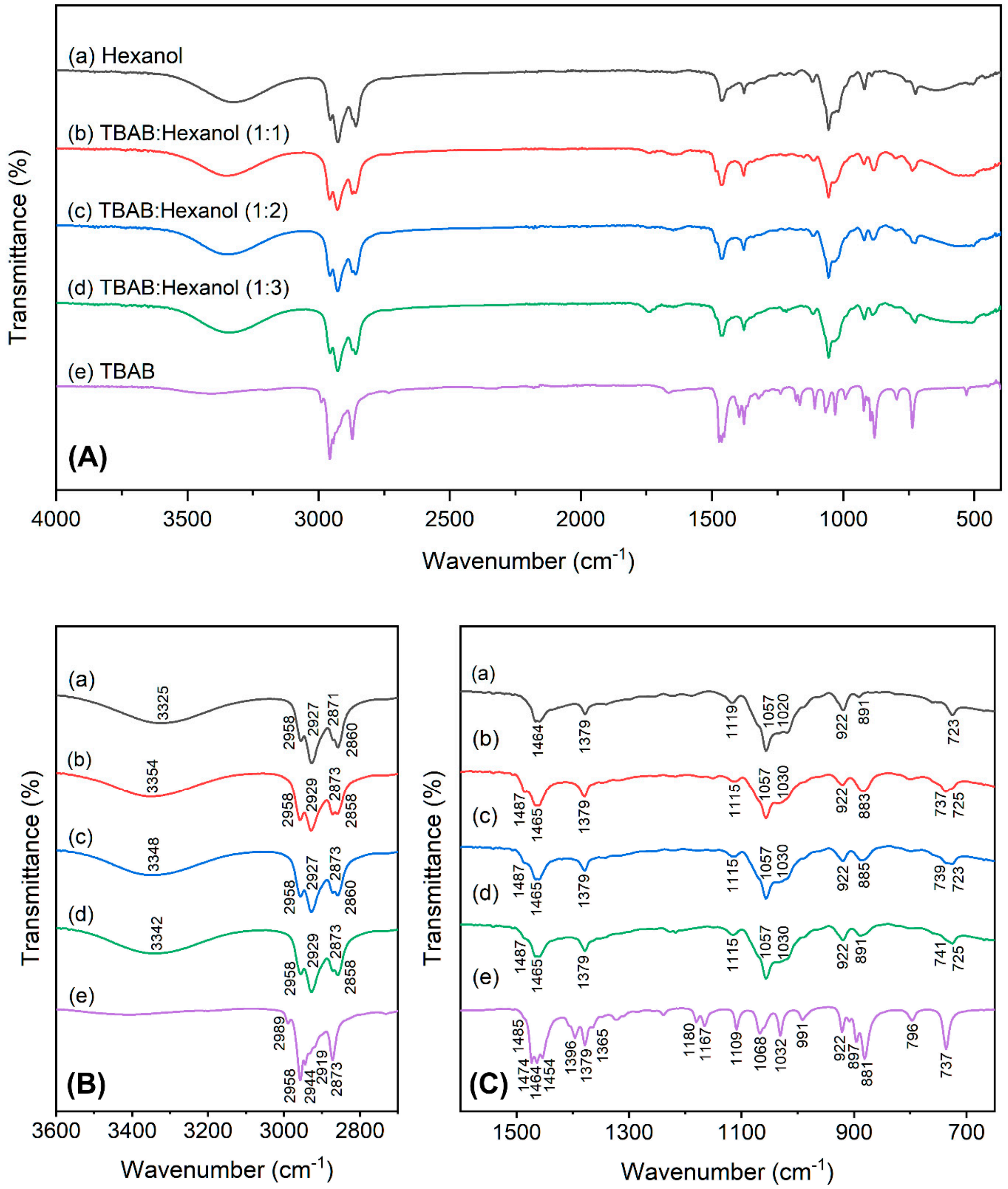

Figure S5. FT-IR spectra of hexanol (a), TBAB (e), and DESs composed of TBAB and hexanol in molar ratios of 1:1 (b), 1:2 (c), and 1:3 (d): (A) full spectra; (B) zoomed region of 2700–3600 cm^{-1}; (C) zoomed region of 650–1600 cm^{-1}.

Table S1. Assignment of specific vibrations to the selected peaks in the FTIR spectrum of hexanol.*

Wavenumber, cm^{-1}	Predominant vibrations
3325	ν(OH), O–H stretching vibrations
2958	$\nu_{as}(CH_3)$, antisymmetric C–H stretching of the methyl group
2927	$\nu_{as}(CH_2)$, antisymmetric C–H stretching of methylene groups
2871	$\nu_s(CH_3)$, symmetric C–H stretching of the methyl group
2860	$\nu_s(CH_2)$, symmetric C–H stretching of methylene groups
1464	$\delta(CH_2)$ + $\delta(CH_3)$, scissoring H–C–H bending of methylene groups and antisymmetric H–C–H bending of the methyl group
1379	$\omega(CH_2)$ + $\omega(CH_3)$, wagging H–C–H bending of methylene and methyl groups
1119	δ(C–O–H), bending of the H_2C–O–H group
1057	ν(C–OH), C–O bond stretching vibration of the H_2C–OH fragment
1020	$\nu(CH_2–CH_2)$, C–C bond stretching vibrations
922	ν_s(C–C–C), symmetric C–C–C stretching of the carbon chain
891	$\nu_s(C–C–CH_3)$, symmetric C–C–CH_3 stretching of the carbon chain
723	$\rho(CH_2)$, rocking H–C–H bending of methylene groups

* ν_s – symmetric bond stretching vibration; ν_{as} – antisymmetric bond stretching vibration; δ – symmetric bond stretching vibration; δ – scissoring bending vibration; ρ – rocking bending vibration; ω – wagging bending vibration.

Table S2. Assignment of specific vibrations to the selected peaks in the FTIR spectrum of DES with TBAB: hexanol ratio equals 1:1.*

Wavenumber, cm^{-1}	Predominant vibrations
3354	ν(OH), O–H stretching vibrations
2958, 2929	$\nu_{as}(CH_2)$ + $\nu_{as}(CH_3)$, antisymmetric C–H stretching of methylene and methyl groups
2873, 2858	$\nu_s(CH_2)$ + $\nu_s(CH_3)$, symmetric C–H stretching of methylene and methyl groups
1487, 1465	$\delta(CH_2)$ + $\delta(CH_3)$, scissoring H–C–H bending of methylene groups and antisymmetric H–C–H bending of the methyl group
1379	$\omega(CH_2)$ + $\omega(CH_3)$, wagging H–C–H bending of methylene and methyl groups
1115	δ(C–O–H), bending of the H_2C–O–H group
1057	ν(C–OH), C–O bond stretching vibration of the H_2C–OH fragment
1030	$\nu(CH_2–CH_2)$, C–C bond stretching vibrations
922	ν_s(C–C–C), symmetric C–C–C stretching of the carbon chain
883	$\nu_s(C–C–CH_3)$, symmetric C–C–CH_3 stretching of the carbon chain
737, 723	$\rho(CH_2)$, rocking H–C–H bending of methylene groups

* ν_s – symmetric bond stretching vibration; ν_{as} – antisymmetric bond stretching vibration; δ – symmetric bond stretching vibration; δ – scissoring bending vibration; ρ – rocking bending vibration; ω – wagging bending vibration.

Table S3. Assignment of specific vibrations to the selected peaks in the FTIR spectrum of DES with TBAB: hexanol ratio equals 1:2.*

Wavenumber, cm^{-1}	Predominant vibrations
3348	ν(OH), O–H stretching vibrations
2958, 2927	$\nu_{as}(CH_2)$ + $\nu_{as}(CH_3)$, antisymmetric C–H stretching of methylene and methyl groups
2873, 2860	$\nu_s(CH_2)$ + $\nu_s(CH_3)$, symmetric C–H stretching of methylene and methyl groups
1487, 1465	$\delta(CH_2)$ + $\delta(CH_3)$, scissoring H–C–H bending of methylene groups and antisymmetric H–C–H bending of the methyl group
1379	$\omega(CH_2)$ + $\omega(CH_3)$, wagging H–C–H bending of methylene and methyl groups
1115	δ(C–O–H), bending of the H_2C–O–H group
1057	ν(C–OH), C–O bond stretching vibration of the H_2C–OH fragment
1030	$\nu(CH_2–CH_2)$, C–C bond stretching vibrations
922	ν_s(C–C–C), symmetric C–C–C stretching of the carbon chain
885	$\nu_s(C–C–CH_3)$, symmetric C–C–CH_3 stretching of the carbon chain
739, 723	$\rho(CH_2)$, rocking H–C–H bending of methylene groups

* ν_s – symmetric bond stretching vibration; ν_{as} – antisymmetric bond stretching vibration; δ – symmetric bond stretching vibration; δ – scissoring bending vibration; ρ – rocking bending vibration; ω – wagging bending vibration.

Table S4. Assignment of specific vibrations to the selected peaks in the FTIR spectrum of DES with TBAB: hexanol ratio equals 1:3.*

Wavenumber, cm^{-1}	Predominant vibrations
3342	ν(OH), O–H stretching vibrations
2958, 2929	$\nu_{as}(CH_2)$ + $\nu_{as}(CH_3)$, antisymmetric C–H stretching of methylene and methyl groups
2873, 2858	$\nu_s(CH_2)$ + $\nu_s(CH_3)$, symmetric C–H stretching of methylene and methyl groups
1487, 1465	$\delta(CH_2)$ + $\delta(CH_3)$, scissoring H–C–H bending of methylene groups and antisymmetric H–C–H bending of the methyl group
1379	$\omega(CH_2)$ + $\omega(CH_3)$, wagging H–C–H bending of methylene and methyl groups
1115	δ(C–O–H), bending of the H_2C–O–H group
1057	ν(C–OH), C–O bond stretching vibration of the H_2C–OH fragment
1030	$\nu(CH_2–CH_2)$, C–C bond stretching vibrations
922	ν_s(C–C–C), symmetric C–C–C stretching of the carbon chain
891	$\nu_s(C–C–CH_3)$, symmetric C–C–CH_3 stretching of the carbon chain
741, 725	$\rho(CH_2)$, rocking H–C–H bending of methylene groups

* ν_s – symmetric bond stretching vibration; ν_{as} – antisymmetric bond stretching vibration; δ – symmetric bond stretching vibration; δ – scissoring bending vibration; ρ – rocking bending vibration; ω – wagging bending vibration.

Table S5. Assignment of specific vibrations to the selected peaks in the FTIR spectrum of TBAB.*

Wavenumber, cm^{-1}	Predominant vibrations
2989, 2958 2944	$\nu_{as}(CH_2)$ + $\nu_{as}(CH_3)$, antisymmetric C–H stretching of methylene and methyl groups
2919, 2873	$\nu_s(CH_2)$ + $\nu_s(CH_3)$, symmetric C–H stretching of methylene and methyl groups
1454–1485	$\delta(CH_2)$ + $\delta(CH_3)$, scissoring H–C–H bending of methylene groups and antisymmetric H–C–H bending of the methyl group
1396, 1379, 1365	$\omega(CH_2)$ + $\omega(CH_3)$ + $\tau(CH_2)$ + $\tau(CH_3)$, wagging and twisting H–C–H bending of methylene and methyl groups
1180, 1167	δ(CNC) + ω(CNC), scissoring and wagging C–N–C bending
1109, 1068, 1032, 991	$\nu(CH_2–CH_2)$ + $\nu(N–CH_2)$, C–C and N–C bonds stretching vibrations
922	ν_s(C–C–C), symmetric C–C stretching of the carbon chain
897	$\nu_s(C–C–CH_3)$, symmetric C–CH_3 stretching of the carbon chain
881	ν_s(N–C–C), symmetric N–C–C stretching
796	$\nu(N–CH_2)$ + $\tau(N–CH_2)$, N–C bonds stretching and torsion C–N–C–C vibrations
737	$\rho(CH_2)$, rocking H–C–H bending of methylene groups

* ν_s – symmetric bond stretching vibration; ν_{as} – antisymmetric bond stretching vibration; δ – symmetric bond stretching vibration; δ – scissoring bending vibration; ρ – rocking bending vibration; ω – wagging bending vibration; τ – twisting bending vibration.
